# Clinician-Performed Bedside Ultrasound in Improving Diagnostic Accuracy in Patients Presenting to the ED with Acute Dyspnea

**DOI:** 10.5811/westjem.2017.1.31223

**Published:** 2017-03-03

**Authors:** Dimitrios Papanagnou, Michael Secko, John Gullett, Michael Stone, Shahriar Zehtabchi

**Affiliations:** * Thomas Jefferson University, Department of Emergency Medicine, Philadelphia, Pennsylvania; † The State University of New York, Downstate Medical Center, Department of Emergency Medicine, Brooklyn, New York; ‡ University of Alabama at Birmingham, Department of Emergency Medicine, Birmingham, Alabama; § Brigham and Women’s Hospital of Harvard Medical School, Department of Emergency Medicine, Boston, Massachusetts

## Abstract

**Introduction:**

Diagnosing acute dyspnea is a critical action performed by emergency physicians (EP). It has been shown that ultrasound (US) can be incorporated into the work-up of the dyspneic patient; but there is little data demonstrating its effect on decision-making. We sought to examine the impact of a bedside, clinician-performed cardiopulmonary US protocol on the clinical impression of EPs evaluating dyspneic patients, and to measure the change in physician confidence with the leading diagnosis before and after US.

**Methods:**

We conducted a prospective observational study of EPs treating adult patients with undifferentiated dyspnea in an urban academic center, excluding those with a known cause of dyspnea after evaluation. Outcomes: 1) percentage of post-US diagnosis matching final diagnosis; 2) percentage of time US changed providers’ leading diagnosis; and 3) change in physicians’ confidence with the leading diagnosis before and after US. An US protocol was developed and standardized prior to the study. Providers (senior residents, fellows, attendings) were trained on US (didactics, hands on) prior to enrollment, and were supervised by an US faculty member. After patient evaluation, providers listed likely diagnoses, documenting their confidence level with their leading diagnosis (scale of 1–10). After US, providers revised their lists and their reported confidence level with their leading diagnosis. Proportions are reported as percentages with 95% confidence interval (CI) and continuous variables as medians with quartiles. We used the Wilcoxon signed-rank test and Cohen’s kappa statistics to analyze data.

**Results:**

A total of 115 patients were enrolled (median age: 61 [51, 73], 59% female). The most common diagnosis before US was congestive heart failure (CHF) (41%, 95%CI, 32–50%), followed by chronic obstructive pulmonary disease (COPD) and asthma. CHF remained the most common diagnosis after US (46%, 95%CI, 38–55); COPD became less common (pre-US, 22%, 95%CI, 15–30%; post-US, 17%, 95%CI, 11–24%). Post-US clinical diagnosis matched the final diagnosis 63% of the time (95%CI, 53–70%), compared to 69% pre-US (95%CI, 60–76%). Fifty percent of providers changed their leading diagnosis after US (95%CI, 41–59%). Overall confidence of providers’ leading diagnosis increased after US (7 [6, 8]) vs. 9 [8, 9], p: 0.001).

**Conclusion:**

Bedside US did not improve the diagnostic accuracy in physicians treating patients presenting with acute undifferentiated dyspnea. US, however, did improve providers’ confidence with their leading diagnosis.

## INTRODUCTION

Dyspnea, the perception of the inability to breathe comfortably, is one of the most common presenting patient complaints in the emergency department (ED).^1^ Patients with dyspnea pose difficult challenges in diagnosis and management to the acute care clinician, as the differential diagnosis for this complaint is broad and varied. Emergency physicians (EP) are often required to make rapid diagnoses and treatment plans for these patients. Because time is of the essence in patients with this critical chief complaint, their presentation requires an aggressive and precise approach.

The initial management for undifferentiated dyspnea includes a history, physical examination, electrocardiogram (ECG), and chest radiograph. This diagnostic approach has been shown to have only intermediate accuracy, identifying the cause of dyspnea in only about two thirds of patients.^2^ Point-of-care ultrasound (POCUS) in the ED is quickly accessible during acute situations, and there is growing evidence to support the role of thoracic US in facilitating an accurate diagnose in dyspneic patients.^3^,^4^ The ability of US to discern between cardiac and non-cardiac etiologies for the source of dyspnea has also been demonstrated.^5^–^7^ There is little data, however, to describe bedside US’s direct effect on physician decision-making in the acute care of the dyspneic patient.

The authors examined the impact of a bedside, clinician-performed cardiopulmonary US protocol on the clinical impression of EPs evaluating patients with dyspnea. The authors hypothesized that integrating such a protocol into the diagnostic work-up of the dyspneic patient would increase the accuracy of EPs’ initial diagnosis, facilitate physicians’ decision-making during patient evaluations, and increase physicians’ confidence level with their leading diagnosis.

## METHODS

This was a prospective observational study of EPs caring for a convenience sample of adult patients who presented to an urban academic medical center with undifferentiated dyspnea. Physicians assigned as the primary EP to a dyspneic patient > 18 years of age were approached by the study investigators and enrolled in the study. Enrollment and patient screening was performed by the study investigators and supervising US faculty members. The study took place over an eight-month period.

### Inclusion

Physicians enrolled included senior (i.e., postgraduate year [PGY]-3 and -4) emergency medicine (EM) residents, fellows, or attending physicians. Physicians were identified after they were assigned to an ED patient with a chief complaint indicating dyspnea (i.e., “shortness of breath”); or with objective signs of dyspnea, specifically tachypnea (respiratory rate > 20 breaths per minute), hypoxemia (pulse oximetry < 94% on room air), or obvious signs of respiratory distress on triage, as noted by the triage nurse in his/her note in the electronic medical record. Patients in this study were of sufficient diagnostic uncertainty; if their pathology was immediately discernable after initial evaluation (i.e., acute asthma exacerbation), they were excluded, as described below.

**Population Health Research Capsule**

What do we already know about this issue?

There is growing evidence to support the role of point-of-care ultrasound (POCUS) in the diagnostic work-up of patients presenting to the ED with acute dyspnea.

What was the research question?

Does a bedside US protocol impact physicians’ diagnosis and confidence level when evaluating dyspneic patients?

What was the major finding of the study?

US did not improve diagnostic accuracy, but did improve physicians’ confidence level with their leading diagnosis.

How does this improve population health?

In dyspneic patients with only mild-to-moderate disease, US may not be as diagnostically impactful on the clinical impression of ED providers compared to patients with higher disease severity.

### Exclusion

Physicians excluded from the study were those providers assigned to one of the following patients: a patient referred from a clinic or office with a known diagnosis; a patient transferred from an outside facility with a known diagnosis; or a patient with a known cause of dyspnea immediately after initial evaluation. The cause of dyspnea for patients was considered to be “known” if patients endorsed the etiology of their symptoms (i.e., asthma exacerbation typical with previous exacerbations), or if patients arrived to the ED with documentation and/or diagnostic results suggesting a diagnosis for their symptoms.

### Study Protocol

Per study protocol, EPs first performed a history and physical examination, and reviewed an ED-performed ECG during an untimed initial evaluation of the dyspneic patient. Baseline patient information was also collected; this included patient demographics, presenting symptoms, symptom onset, patient-reported dyspnea severity (Likert scale from 1 [“not short of breath”] to 10 [“very short of breath”]), vital signs, and past medical history. Following initial patient evaluation, physicians were surveyed and asked to select and rank the most likely diagnoses for their patient’s dyspnea from a list of possible diagnoses to choose from, as well as to document their confidence level with their leading diagnosis (scale of 1–10). Physicians then performed a supervised focused point-of-care cardiopulmonary US on their dyspneic patient. On completion of the US exam, providers were surveyed for a second time, allowing them to revise their differential diagnoses and confidence levels. A chest radiograph was not part of the initial untimed work-up; therefore, a radiograph was not reviewed prior to the bedside cardiopulmonary US exam.

### Outcomes

Outcomes of the study included the following: 1) percentage of the post-US diagnosis matching the final diagnosis, which was determined by blinded chart review (i.e., two investigators separately reviewed patients’ charts and determined the final diagnoses; discrepancies were solved by a third investigator’s blinded review); 2) percentage of time US changed the leading diagnosis determined by the provider; and 3) change in physician confidence level with the leading diagnosis before and after US by surveying the provider.

### Ultrasound Protocol

The US protocol was developed and standardized prior to study. The protocol consisted of at least two views of the heart (parasternal long, parasternal short, subxiphoid, apical views), as well as anterior and posterolateral views of the lungs bilaterally. While enrolled providers were encouraged to obtain all of the aforementioned views, they were required to acquire US images that would assist them in answering specific point-of-care questions for their dyspneic patients. Based on images acquired, providers were asked the following questions: to describe left ventricular (LV) wall motion (i.e., depressed, normal, hyperdynamic); to identify the presence of a pericardial effusion and, if present, to describe its size (i.e., small moderate, large); to identify the presence of right ventricular (RV) strain (i.e., RV dilatation or a D-shaped LV); to identify lung A lines and/or B lines by location (i.e., right anterior, right posterolateral, left anterior, left posterolateral); to identify the presence or absence of a [right and/or left] pulmonary effusion; and to identify the presence of lung sliding bilaterally. With specific regards to lung US, providers were asked to qualitatively report if there was a predominance of an A-line or B-line pattern for lung images acquired; providers were not asked to quantify the number of B-lines appreciated. Supervising US faculty recorded the US findings, which were verbalized by the provider performing the patient US.

Providers (PGY-3 or -4, fellows, attendings) were trained on US (didactics and hands-on) prior to enrollment, and were supervised by the enrolling US faculty member for quality assurance and recording purposes. Supervising US faculty members did not provide feedback during scanning, and did not influence the EP’s image interpretation. Upon completion of the second provider survey, supervising US faculty members then discussed patient US images with respective providers to ensure salient findings were noted so as to not compromise and/or delay patient care.

### Ultrasound Training

US training is provided to all residents, fellows, and faculty in the department. EM residents undergo a two-week US rotation at some point during the first half of their curriculum. Cardiac and lung US scans are taped during quality assurance review sessions.. While on rotation, each resident receives at least two hours of didactics dedicated to cardiac and pulmonary applications of US, and are required to complete a minimum of 15 cardiac and thoracic quality scans. Residents’ procedural competency is supervised and assessed by the US rotation director; those residents who do not demonstrate competency are remediated until procedural competency is achieved. US fellows are credentialed early during their fellowship to perform cardiac and non-cardiac scans according to American College of Emergency Physician (ACEP) guidelines. Faculty members in the department attend biannual US skills training workshops, offered by credentialed US faculty members within the department, where cardiac and lung US skills are reinforced and assessed for procedural competency.

Knowledge of POCUS skills (i.e., image recognition, identification of pathologic findings) was demonstrated prior to enrollment, by achieving a score of >80% on the cardiac and lung portions of the ACEP online US examination, available at: http://emsono.com/acep. In addition, all enrollees received a brief 15-minute refresher bedside training session on cardiac and lung US views. The US examination and refresher session were complete for all residents, fellows, and faculty before initiating enrollment in the ED where the study was performed.

### Ultrasound Machines and Quality Assurance

A SonoSite portable M-Turbo ultrasound unit and a GE LOGIQ P5 machine were used for the study. US examinations were performed using a 5-2 MHz curved-array transducer for lung studies, and a 5-1 MHz phased-array transducer for cardiac studies. All US images and clips were saved and recorded onto the US system, and reviewed by emergency US fellowship-trained faculty at weekly departmental performance improvement / quality assurance tape review sessions.

### Consent

Study investigators obtained written informed consent before physician enrollment. Investigators offered a detailed explanation of the study’s objectives and benefits, and answered any questions prior to enrollment. All physician subjects had the right to refuse enrollment into the study. Patients were provided with an information sheet that described the study. The institutional review board approved the study.

### Statistical Analysis

The study was powered based on initial pilot data that the US protocol would result in a change in differential diagnosis in 75% of cases. We aimed to demonstrate this with a 95% CI precision of 10%, which necessitated at least 73 physician-patient ED encounters of acute dyspnea be enrolled using a binomial exact calculation.^8^ Proportions are reported as percentages with 95% CI, calculated by the Agresti-Coull method; and continuous variables are reported as medians with quartiles. We used the Wilcoxon signed-rank test and Cohen’s kappa statistics to analyze data.

To facilitate comparisons between pre- and post-US diagnoses with the final diagnosis, we categorized diagnoses into three super-types: cardiac (i.e., congestive heart failure [CHF], pulmonary (i.e., chronic obstructive pulmonary disease), and other (i.e., anemia). To avoid multiple comparisons, similar diagnoses were categorized into these three super-type categories using weighted Cohen’s kappa statistics. We used Cohen’s kappa to measure the concordance between pre- and post-US diagnoses with the final diagnosis across cardiac, pulmonary, and other diagnoses.

## RESULTS

We included a total of 115 physician-patient encounters of patients presenting to the ED with dyspnea in the study. Patients were 59% female; and the median age was 61 years [51, 73]. Almost all patients (99%) provided a triage complaint of “shortness of breath” with 54 (47%) presenting with symptoms of dyspnea for less than 24 hours. The most common self-reported comorbid conditions in participants’ past medical histories were hypertension (67%), diabetes (44%) and CHF (29%) (Table 1.

All patients underwent POCUS assessment conducted by the enrolled physician, under the supervision of a credentialed US faculty member. Twenty-seven physicians in total participated in the study, which included third- and fourth-year EM residents; ultrasound fellows; and EM faculty members. Of the ultrasounds performed, 23 were performed by PGY-3 residents (20%); 31 were performed by PGY-IV residents (27%); 20 were performed by US fellows (17%); and 41 were performed by faculty members (36%). Providers were able to obtain all four cardiac views and all four lung views in 93% of cases (107 out of 115 physician-patient encounters).

The top seven diagnostic conditions, and their respective proportions, are presented in Table 2, along with the final diagnosis. Overall, CHF was the most common diagnosis before US (47%, 95% CI [32%–50%]), followed by COPD and asthma. CHF remained the most common diagnosis after US (46%, 95% CI [37%–56%]), while COPD became less common after ultrasonographic assessment (pre-US, 22%, 95% CI [15%–30%]; post-US, 17%, 95% CI [11%–24%]) (Table 2). Post-US clinical diagnosis matched final diagnosis 63% of the time (95% CI [53%–70%]), compared to 69% pre-US with the final diagnosis (95% CI [60%–76%]).

Survey of enrolled physicians demonstrated that US narrowed the differential diagnosis in 78% of cases. There was a change in the leading diagnosis post-US in 32% of cases, while the diagnosis remained the same in 68% of cases. One out of two physicians reported that the incorporation of bedside US into the patient management changed the diagnosis and/or treatment plan (Table 3). After completion of the US protocol, providers’ lists of differential diagnoses narrowed (or decreased) by one diagnosis (median change, −1, p<0.001, via the Wilcoxon signed-rank test).

Providers’ confidence level with their leading diagnoses increased after the US protocol. The median pre-US confidence level was 7 out of 10, compared to 9 out of 10 post-US; the median +2 change suggesting increased confidence was statistically significant by the Wilcoxon signed-rank test (p<0.001). Sub-analysis of the 79 cases where the leading pre-US diagnosis remained the same post-US also demonstrated an increased in confidence level, 8 out of 10 to 9 out of 10, respectively (p<0.001).

Agreement of physicians’ leading diagnoses before and after US with the final diagnosis, as determined by the Cohen’s kappa statistic, was moderate. Agreement with the final primary diagnosis was slightly lower pre-US compared to the post-US diagnosis (Kappa: 0.45 pre-US vs. 0.56 post-US) (Table 4).

## DISCUSSION

The agreement of physicians’ leading diagnosis before and after US with the final diagnosis was moderate, suggesting that bedside US has minimal impact on the clinical evaluation of acute dyspnea; however, we observed a modest increase in physician’s diagnostic confidence, as well as changes in the management in some cases,.

There are several other studies that address the effect of US in differentiating the etiology of acute dyspnea. They differ from this study in that their US protocol is either limited to lung imaging, evaluates for the presence or absence of interstitial syndrome (i.e., typically CHF), or relies on few experienced sonographers. (In certain cases, studies do not provide details of sonographers’ experience levels.) The specific effect on clinical decision-making in a patient’s acute management has not been thoroughly studied.

Goffi et al. studied 50 ED patients with acute undifferentiated dyspnea, and did find a significant diagnostic and therapeutic impact of lung US on management.^9^ Lung US changed the diagnosis in 44% of cases and the management in 58% of cases, a significant difference from our data. They found fair agreement of clinical diagnosis to the final diagnosis (Cohen’s kappa coefficient = 0.25, 0.32, and 0.26 for main, pathophysiologic, and etiological diagnosis, respectively; p <0.01), and excellent agreement between US-assisted diagnosis and final diagnosis (Kappa coefficient = 0.94, 0.84, and 0.81, respectively; p <0.01). This study did not include cardiac imaging, and the number of sonographers and their level of experience were not clearly outlined.

Liteplo et al. proposed the ETUDES (Emergency Thoracic Ultrasound in the Differentiation of the Etiology of Shortness of Breath) exam for undifferentiated dyspnea in 2009.^10^ This application was mainly designed to differentiate between CHF and COPD by counting the number of B-lines in multiple thoracic zones. They studied 94 patients for the possible diagnosis of CHF using an eight-zone lung exam, and found a positive likelihood ratio of 3.88 and a negative likelihood ratio of 0.5.

In 2012 Cibinel et al. attempted to differentiate cardiogenic and non-cardiogenic etiologies of dyspnea in the ED.^11^ They evaluated 56 patients for alveolar interstitial syndrome (AIS) or pleural effusions. They found diffuse AIS to be 93% sensitive and 84% specific for cardiogenic dyspnea. Detection of pleural effusions, however, was not helpful in the differentiation (84% sensitive, 52% specific). Anderson et al. examined the effect of a multi-organ approach to diagnosing acute decompensated heart failure (ADHF) in 2013.^12^ They examined left ventricular ejection fraction, inferior vena cava, and eight thoracic zones on 101 patients. US exams were performed by five expert sonographers. Specificity for diagnosing ADHF was 100%. In another study by Unluer et al., US was placed in the hands of ED nurses who performed the bedside lung ultrasound in emergency (BLUE) protocol on 96 acutely dyspneic ED patients to discern between a cardiac versus respiratory underlying etiology.^13^,^14^ Agreement with the final diagnosis was 0.917; sensitivity and specificity were 95.35% and 95.74%, respectively. A study by Kajimoto et al. in 2012 did include lung, cardiac, and inferior vena cava US in the evaluation of acute dyspnea in 90 patients.^15^ They demonstrated a sensitivity, specificity, negative predictive value, and positive predictive value of 94.3%, 91.9%, 91.9%, and 94.3%, respectively, for the diagnosis of acute decompensated CHF.

Appraisal of the literature demonstrates that bedside cardiopulmonary ultrasonography has the capacity to gather accurate diagnostic information in the acutely dyspneic patient for the purpose of narrowing the differential diagnosis, if not helping arrive at the specific diagnosis. What has not been definitively demonstrated by the evidence is whether bedside US performs better than the standard clinician evaluation. The data in our study do not support this practice. In fact, in contrast to prior studies, our results imply that US can decrease diagnostic accuracy for acute undifferentiated dyspnea. This may indicate that cardiopulmonary US requires significant experience to use accurately. Primarily, the clinicians in this study were not US specialists. There was significant variability in US skill and experience among sonographers; subgroup analyses may yield different results when stratified by sonographer experience.

There is little doubt that cardiopulmonary sonography yields objective signs of specific pathology.^16^–^22^ B-lines, for example, are a well-defined sign of interstitial syndrome; however, the number and severity of B-lines, as well as their clinical significance, may elicit subjective interpretations. Some US applications have inherently greater inter-rater variability in interpretation. Thus, some applications may be classified as advanced, requiring greater experience and understanding than others to interpret and accurately apply in clinical scenarios.

An important point to consider in this investigation is that the most critically ill dyspneic patients were not enrolled in the study. Table 1 shows that 56 patients (54% of all patients enrolled) rated their dyspnea severity an 8 or higher on a 10-point Likert scale. Previous studies have highlighted the positive impact US has had on the clinical impression of critically ill dyspneic patients. Our results suggest that US may not have a similar diagnostic impact in patients with mild-to-moderate disease. Furthermore, given the variability of US training level in the physician providers enrolled, the use of POCUS for patients with mild-to-moderate dyspnea severity may not be as helpful as suggested in previous studies with higher percentages of dyspneic patients with severe cases of disease.

The implication for clinical practice is that bedside cardiopulmonary US may be useful and change patient management. Findings may be misleading and should not be used without proper training and adequate awareness of the limitations of both imaging and sonographer. ECG and chest radiography, for example, are operator dependent as well. It should be emphasized here that the integration of bedside US in the hands of non-specialists, as well as the degree of training required for this purpose, remains to be determined.

While no definitive conclusions can be drawn, the study does raise questions for further consideration and investigation. Results imply that US may only be accurate for specific diagnoses in experienced hands, while its ability to narrow a differential diagnosis may be possible for sonographers of varied skill levels. This study suggests that in the hands of non-US specialists, US may be diagnostically misleading. Larger studies on clinical bedside US’s influence on non-expert physician-sonographer’s decision-making process would be valuable to investigate this claim.

Prospective studies should randomize patients to diagnostic treatment arms supplemented with POCUS, similar to the strategy executed by Laursen et al. in 2014.^23^ In their study, dyspneic patients were enrolled by both vital signs and symptoms, and were randomly assigned to a standard diagnostic strategy (control group) versus standard diagnostic strategy with POCUS imaging of the heart, lungs, and deep veins of the lower extremities (treatment group). Furthermore, prospective studies should include severely dyspneic patients who cannot provide a history and/or consent, or who require emergent endotracheal intubation; these considerations would potentially demonstrate US’s diagnostic efficacy.

## LIMITATIONS

There are several study limitations worth noting. A subgroup analysis of specific factors may refine the study’s results; these include patient severity of disease on presentation and the variable experience level of the sonographers. Determining the specific US view(s) (i.e., specific cardiac and/or lung windows) or US finding(s) that had the greatest influence on the diagnosis may clarify the value of the study’s US protocol. It is likely that severe disease will be more obvious on US examination, compared to mild to moderate disease. It is also likely that less pre- to post-US change is found in dyspneic patients with obvious clinical presentations, where the diagnosis is suspected before the US; it is in patients where there is significant diagnostic uncertainty where the US protocol has significant potential to change provider’s diagnosis and their confidence in their diagnosis.

A subgroup analysis of specific US images (read later by experienced emergency US faculty) would clarify whether equivocal results were a result of incorrect image interpretation. It would also determine whether the clinical bedside environment influenced image interpretation. This analysis was not performed. Furthermore, patients may have had multiple co-diagnoses (i.e., COPD and CHF), which may have confounded the results. The study is also biased toward mildly to moderately dyspneic patients with potentially more subtle pathologies. Larger numbers would be needed to further elucidate the effect of these limitations.

## CONCLUSION

Bedside US did not improve the diagnostic accuracy in physicians treating ED patients with acute undifferentiated dyspnea in the present study. The incorporation of clinician-performed bedside US for acutely dyspneic patients, however, may help narrow the clinician’s differential diagnoses and change the diagnostic and/or therapeutic plan in half of cases. Bedside US did not affect the actual diagnosis based on the clinical assessment and US imaging when compared to the final diagnosis; however, larger studies on clinical outcomes and decision-making effects of clinician-performed bedside US for patients with acute dyspnea may be necessary to further elucidate the trends suggested by our data. Results suggest that in dyspneic patients with mild-to-moderate severity of disease, cardiopulmonary US may not be as diagnostically impactful on the clinical impression of the provider when compared to studies of patients with higher severity of disease.

## Figures and Tables

**Table 1:** Baseline patient demographics and characteristics in study of the usefulness of bedside ultrasound in diagnosing dyspnea.

Variables	N = 115	%	Median (quartiles)
Demographics			
Female gender	68	59%	--
Age (median, quartiles)	115	--	61 (51, 73)
Presenting symptoms			
Shortness of breath	114	99%	--
Chest pain	8	7%	--
Edema	4	4%	--
Symptom onset			
<24 Hours	54	47%	--
1–7 Days	32	28%	--
>7 Days	29	25%	--
Dyspnea severity*			
Overall (median, quartiles)	104	--	8 (6, 9)
2–5	19/104	18%	--
6–7	29/104	28%	--
8–10	56/104	54%	--
Vital signs (median, quartiles)			
SBP (mmHg)	115	--	144 (122, 167)
DBP (mmHg)	115	--	83 (76, 96)
Heart rate (beats/min)	115	--	87 (76, 106)
Respiratory rate (breaths/min)	115	--	22 (18, 26)
Oxygen saturation (%)	113	--	98 (96, 100)
Temperature (degrees F)	112	--	98 (98, 99)
Past medical history			
Diabetes	51	44%	--
Hypertension	77	67%	--
Renal	21	18%	--
CHF	33	29%	--
CAD	31	27%	--
Asthma	24	21%	--
COPD	21	18%	--
Smoker	13	11%	--
DVT/PE	4	4%	--
Cancer	5	4%	--
Other	61	53%	--

* Dyspnea severity was reported by the patient, and measured on a visual analogue scale of 1 (mild) to 10 (severe).

*SBP,* systolic blood pressure; *DBP,* diastolic blood pressure; *CHF,* congestive heart failure; *CAD,* coronary artery disease; *COPD,* chronic obstructive pulmonary disease; *DVT,* deep vein thrombosis; *PE,* pulmonary embolism

**Table 2:** Comparison of pre- and post-ultrasound diagnostic categories to final hospital diagnosis.

Leading diagnosis	Pre-US	Post-US	Final diagnosis
	
	N (%, 95% CI)	N (%, 95% CI)	
All diagnoses	Total N=115	Total N=115	Total N=115
ACS	4 (4%, 1 to 9%)	4 (4%, 1 to 9%)	7 (6%, 3 to 12%)
CHF	47 (41%, 32 to 50%)	53 (46%, 38 to 55%)	39 (34%, 26 to 43%)
Pneumonia	5 (4%, 2 to 10%)	8 (7%, 3 to 13%)	5 (4%, 2 to 10%)
Asthma/reactive airway disease	14 (12%, 7 to 20%)	8 (7%, 3 to 13%)	12 (10%, 16 to 18%)
COPD	25 (22%, 15 to 30%)	19 (17%, 11 to 24%)	24 (21%, 14 to 29%)
PE	11 (10%, 5 to 16%)	9 (8%, 4 to 14%)	1 (1%, 0 to 5%)
Other	9 (8%, 4 to 14%)	14 (12%, 7 to 20%)	27 (23%, 17 to 32%)
Physicians’ confidence level^*^ (Median, quartile)	7 (6, 8)	9 (8, 9)	---

*US,* ultrasound; *CI,* confidence interval; *ACS,* acute coronary syndrome; *CHF,* congestive heart failure; *COPD,* chronic obstructive pulmonary disease; *PE,* pulmonary embolism

**Table 3:** Impact of ultrasound on physicians’ differential diagnosis and confidence level before and after the ultrasound.

Impact of ultrasound	n/N	95% CI
Narrowed differential diagnosis list	90/115	78% (70 to 85%)
Changed leading diagnosis	37/115	32% (24 to 42%)
Change in confidence level*	2 (1, 2)	-
Pre-US diagnosis matched final diagnosis	79/115	69% (60 to 76%)
Post-US diagnosis matched final diagnosis	72/115	63% (53 to 70%)
Overall change in diagnosis and/or treatment**	58/115	50% (41 to 59%)

*US,* ultrasound; *CI,* confidence interval

* Determined by calculating the difference in physicians’ confidence level in their leading diagnosis before and after the ultrasound.

** Determined by surveying the treating physicians.

**Table 4:** Agreement of leading diagnoses (cardiac vs. pulmonary vs. other) before and after the ultrasound with the final diagnosis.

Clinical impression	Kappa	95% CI
Pre-US vs. final diagnosis	0.45	0.31 to 0.58
Post-US vs. final diagnosis	0.56	0.43 to 0.69

*US,* ultrasound; *CI*, confidence interval
